# 6-Fluoro-1*H*-indole-3-carb­oxy­lic acid

**DOI:** 10.1107/S1600536812016935

**Published:** 2012-04-28

**Authors:** Ming Lou, Yang-Hui Luo

**Affiliations:** a College of Chemistry and Chemical Engineering, Southeast University, Nanjing 211189, People’s Republic of China

## Abstract

In the title compound, C_9_H_6_FNO_2_, all the non-H atoms are approximately coplanar, the carb­oxy O atoms deviating by 0.0809 and −0.1279 Å from the indole plane. In the crystal, O—H⋯O hydrogen bonds link the mol­ecules into dimers which are linked *via* N—H⋯O hydrogen bonds and π–π inter­actions [centroid–centroid distance = 3.680 (2) Å]

## Related literature

For the origin of the material studied, see: Kunzer & Wendt (2011 ▶). For a related structure, see: Luo *et al.* (2011 ▶).
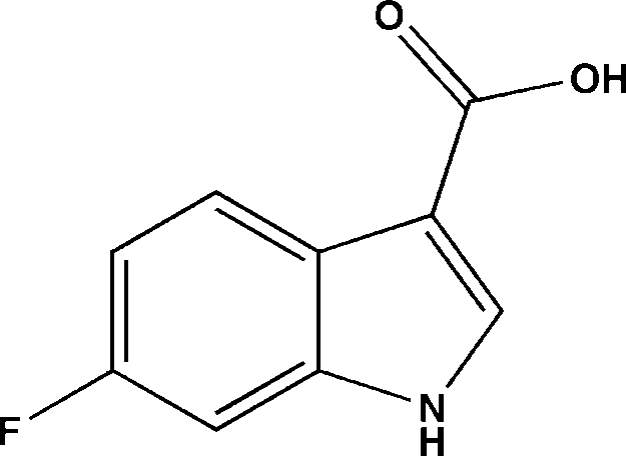


## Experimental

### 

#### Crystal data

C_9_H_6_FNO_2_
*M* *_r_* = 179.15
Monoclinic, 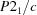

*a* = 7.0054 (14) Å
*b* = 11.699 (2) Å
*c* = 9.2947 (19) Å
β = 104.15 (3)°
*V* = 738.7 (3) Å^3^
*Z* = 4
Mo *K*α radiation
μ = 0.13 mm^−1^
*T* = 293 K
0.3 × 0.3 × 0.2 mm

#### Data collection

Rigaku SCXmini diffractometer
Absorption correction: multi-scan (*CrystalClear*; Rigaku, 2005 ▶) *T*_min_ = 0.961, *T*_max_ = 0.974
7541 measured reflections
1693 independent reflections
1418 reflections with *I* > 2σ(*I*)
*R*_int_ = 0.033

#### Refinement

*R*[*F* ^2^ > 2σ(*F* ^2^)] = 0.043
*wR*(*F* ^2^) = 0.115
*S* = 1.08
1693 reflections
123 parameters
H atoms treated by a mixture of independent and constrained refinement
Δρ_max_ = 0.21 e Å^−3^
Δρ_min_ = −0.21 e Å^−3^

### 

Data collection: *CrystalClear* (Rigaku, 2005 ▶); cell refinement: *CrystalClear*; data reduction: *CrystalClear*; program(s) used to solve structure: *SHELXS97* (Sheldrick, 2008 ▶); program(s) used to refine structure: *SHELXL97* (Sheldrick, 2008 ▶); molecular graphics: *DIAMOND* (Brandenburg & Putz, 2005 ▶); software used to prepare material for publication: *SHELXL97*.

## Supplementary Material

Crystal structure: contains datablock(s) I, global. DOI: 10.1107/S1600536812016935/rn2099sup1.cif

Structure factors: contains datablock(s) I. DOI: 10.1107/S1600536812016935/rn2099Isup2.hkl

Supplementary material file. DOI: 10.1107/S1600536812016935/rn2099Isup3.cml

Additional supplementary materials:  crystallographic information; 3D view; checkCIF report

## Figures and Tables

**Table 1:** Hydrogen-bond geometry (Å, °)

*D*—H⋯*A*	*D*—H	H⋯*A*	*D*⋯*A*	*D*—H⋯*A*
N1—H1⋯O1^i^	0.86 (2)	2.159 (19)	2.8925 (17)	142.8 (17)
O2—H2⋯O1^ii^	0.82	1.78	2.5954 (17)	170

Symmetry codes: (i) 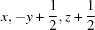; (ii) 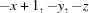.

**Fig. 1.**
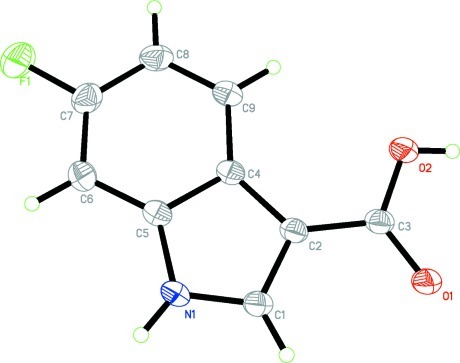
The molecular structure of the title compound with the atom-labelling scheme. Displacement ellipsoids are drawn at the 30% probability level. H atoms are represented as small spheres of arbitrary radii.

**Fig. 2.**
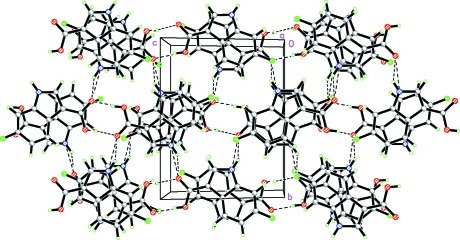
A packing view down the a axis showing the three dimensional network. Intermolecular hydrogen bonds are shown as dashed lines.

## supplementary crystallographic information

### Comment

Indole-3-carboxylic acid and its derivatives are important chemical materials, because they are excellent auxins for plants ( Kunzer & Wendt, 2011) and drug intermediates for many pharmaceutical products (Luo et al., 2011).

The molecular structure of the title compound is shown in Fig. 1. All the non-H atoms are approximately coplanar: the carboxy O atoms deviating by 0.0809 and -0.1279 Å from the indole plane..

In the crystal structure of the title compound, intermolecular O—H···O hydrogen bonds linked the molecules into dimers and the dimers are linked via intermolecular N—H···O hydrogen bonds and π–π interactions [centroid–centroid distance = 3.680 (2) Å] (Fig. 2).

### Experimental

The title compound was purchased commercially from ChemFuture PharmaTech, Ltd (Jiangsu) and used as received without further purification. Crystals of it were obtained by slow evaporation of a methanol solution.

### Refinement

All H atoms attached to C, N and O atoms were fixed geometrically and treated as riding with C—H = 0.93 Å (CH), O—H = 0.82 Å and N—H = 0.86 Å with *U*_iso_(H) = 1.2*U*_eq_(CH), *U*_iso_(H) = 1.35*U*_eq_(N) and *U*_iso_(H) = 1.5*U*_eq_(O).

### Crystal data

**Table:**

C_9_H_6_FNO_2_	*F*(000) = 368
*M**_r_* = 179.15	*D*_x_ = 1.611 Mg m^−^^3^
Monoclinic, *P*2_1_/*c*	Mo *K*α radiation, λ = 0.71073 Å
Hall symbol: -P 2ybc	Cell parameters from 1693 reflections
*a* = 7.0054 (14) Å	θ = 3.5–27.5°
*b* = 11.699 (2) Å	µ = 0.13 mm^−^^1^
*c* = 9.2947 (19) Å	*T* = 293 K
β = 104.15 (3)°	Block, brown
*V* = 738.7 (3) Å^3^	0.3 × 0.3 × 0.2 mm
*Z* = 4	

### Data collection

**Table:**

Rigaku SCXmini diffractometer	1693 independent reflections
Radiation source: fine-focus sealed tube	1418 reflections with *I* > 2σ(*I*)
Graphite monochromator	*R*_int_ = 0.033
Detector resolution: 13.6612 pixels mm^-1^	θ_max_ = 27.5°, θ_min_ = 3.5°
CCD_Profile_fitting scans	*h* = −9→9
Absorption correction: multi-scan (*CrystalClear*; Rigaku, 2005)	*k* = −15→15
*T*_min_ = 0.961, *T*_max_ = 0.974	*l* = −12→12
7541 measured reflections	

### Refinement

**Table:**

Refinement on *F*^2^	Primary atom site location: structure-invariant direct methods
Least-squares matrix: full	Secondary atom site location: difference Fourier map
*R*[*F*^2^ > 2σ(*F*^2^)] = 0.043	Hydrogen site location: inferred from neighbouring sites
*wR*(*F*^2^) = 0.115	H atoms treated by a mixture of independent and constrained refinement
*S* = 1.08	*w* = 1/[σ^2^(*F*_o_^2^) + (0.0591*P*)^2^ + 0.1428*P*] where *P* = (*F*_o_^2^ + 2*F*_c_^2^)/3
1693 reflections	(Δ/σ)_max_ < 0.001
123 parameters	Δρ_max_ = 0.21 e Å^−^^3^
0 restraints	Δρ_min_ = −0.21 e Å^−^^3^

### Special details

**Table:**

Geometry. All e.s.d.'s (except the e.s.d. in the dihedral angle between two l.s. planes) are estimated using the full covariance matrix. The cell e.s.d.'s are taken into account individually in the estimation of e.s.d.'s in distances, angles and torsion angles; correlations between e.s.d.'s in cell parameters are only used when they are defined by crystal symmetry. An approximate (isotropic) treatment of cell e.s.d.'s is used for estimating e.s.d.'s involving l.s. planes.
Refinement. Refinement of *F*^2^ against ALL reflections. The weighted *R*-factor *wR* and goodness of fit *S* are based on *F*^2^, conventional *R*-factors *R* are based on *F*, with *F* set to zero for negative *F*^2^. The threshold expression of *F*^2^ > σ(*F*^2^) is used only for calculating *R*-factors(gt) *etc*. and is not relevant to the choice of reflections for refinement. *R*-factors based on *F*^2^ are statistically about twice as large as those based on *F*, and *R*- factors based on ALL data will be even larger.

### Fractional atomic coordinates and isotropic or equivalent isotropic displacement parameters (Å^2^)

**Table:**

	*x*	*y*	*z*	*U*_iso_*/*U*_eq_	
O2	0.60596 (19)	−0.07115 (8)	0.16978 (12)	0.0489 (3)	
H2	0.5681	−0.0785	0.0795	0.073*	
O1	0.5438 (2)	0.11097 (9)	0.10977 (12)	0.0514 (3)	
F1	0.96927 (19)	−0.13190 (10)	0.89741 (12)	0.0727 (4)	
N1	0.70843 (19)	0.17044 (11)	0.55849 (14)	0.0394 (3)	
C3	0.5997 (2)	0.03550 (12)	0.20426 (16)	0.0377 (3)	
C2	0.6606 (2)	0.06339 (11)	0.35810 (16)	0.0343 (3)	
C4	0.74187 (19)	−0.00793 (11)	0.48256 (15)	0.0330 (3)	
C9	0.7914 (2)	−0.12279 (12)	0.50269 (18)	0.0397 (4)	
H9	0.7736	−0.1718	0.4217	0.048*	
C6	0.8487 (2)	0.02441 (13)	0.74928 (18)	0.0432 (4)	
H6	0.8692	0.0722	0.8316	0.052*	
C8	0.8663 (2)	−0.16262 (13)	0.6426 (2)	0.0465 (4)	
H8	0.8991	−0.2394	0.6583	0.056*	
C5	0.7710 (2)	0.06329 (12)	0.60672 (16)	0.0351 (3)	
C1	0.6428 (2)	0.17026 (12)	0.41164 (16)	0.0382 (3)	
H1A	0.5925	0.2335	0.3542	0.046*	
C7	0.8931 (2)	−0.08866 (15)	0.76066 (19)	0.0475 (4)	
H1	0.702 (3)	0.2305 (17)	0.611 (2)	0.053 (5)*	

### Atomic displacement parameters (Å^2^)

**Table:**

	*U*^11^	*U*^22^	*U*^33^	*U*^12^	*U*^13^	*U*^23^
O2	0.0787 (8)	0.0289 (6)	0.0382 (6)	−0.0018 (5)	0.0127 (6)	−0.0044 (4)
O1	0.0856 (9)	0.0304 (6)	0.0364 (6)	−0.0084 (5)	0.0116 (6)	0.0019 (4)
F1	0.0872 (8)	0.0638 (8)	0.0524 (7)	0.0055 (6)	−0.0111 (6)	0.0161 (5)
N1	0.0514 (7)	0.0270 (6)	0.0399 (7)	−0.0006 (5)	0.0114 (6)	−0.0068 (5)
C3	0.0479 (8)	0.0291 (7)	0.0388 (8)	−0.0050 (6)	0.0156 (6)	−0.0007 (5)
C2	0.0397 (7)	0.0281 (7)	0.0370 (8)	−0.0039 (5)	0.0128 (6)	−0.0025 (5)
C4	0.0321 (6)	0.0291 (7)	0.0393 (8)	−0.0030 (5)	0.0115 (6)	−0.0025 (5)
C9	0.0417 (8)	0.0300 (7)	0.0475 (9)	0.0004 (6)	0.0114 (7)	−0.0042 (6)
C6	0.0450 (8)	0.0430 (8)	0.0388 (8)	−0.0053 (7)	0.0046 (6)	−0.0044 (6)
C8	0.0456 (9)	0.0329 (8)	0.0585 (10)	0.0055 (6)	0.0079 (7)	0.0050 (7)
C5	0.0342 (7)	0.0306 (7)	0.0411 (8)	−0.0034 (5)	0.0101 (6)	−0.0038 (6)
C1	0.0477 (8)	0.0287 (7)	0.0392 (8)	−0.0010 (6)	0.0124 (6)	0.0001 (6)
C7	0.0442 (8)	0.0477 (9)	0.0447 (9)	0.0006 (7)	−0.0005 (7)	0.0089 (7)

### Geometric parameters (Å, º)

**Table:**

O2—C3	1.2916 (17)	C4—C9	1.389 (2)
O2—H2	0.8200	C4—C5	1.3974 (19)
O1—C3	1.2394 (18)	C9—C8	1.360 (2)
F1—C7	1.351 (2)	C9—H9	0.9300
N1—C1	1.330 (2)	C6—C7	1.357 (2)
N1—C5	1.3668 (19)	C6—C5	1.381 (2)
N1—H1	0.86 (2)	C6—H6	0.9300
C3—C2	1.426 (2)	C8—C7	1.373 (3)
C2—C1	1.363 (2)	C8—H8	0.9300
C2—C4	1.427 (2)	C1—H1A	0.9300
			
C3—O2—H2	109.5	C7—C6—C5	115.15 (14)
C1—N1—C5	109.75 (12)	C7—C6—H6	122.4
C1—N1—H1	121.7 (13)	C5—C6—H6	122.4
C5—N1—H1	128.4 (13)	C9—C8—C7	119.56 (15)
O1—C3—O2	122.44 (14)	C9—C8—H8	120.2
O1—C3—C2	120.84 (13)	C7—C8—H8	120.2
O2—C3—C2	116.72 (13)	N1—C5—C6	129.53 (14)
C1—C2—C3	122.98 (14)	N1—C5—C4	107.80 (13)
C1—C2—C4	107.12 (12)	C6—C5—C4	122.67 (14)
C3—C2—C4	129.88 (13)	N1—C1—C2	109.68 (13)
C9—C4—C5	118.97 (13)	N1—C1—H1A	125.2
C9—C4—C2	135.39 (13)	C2—C1—H1A	125.2
C5—C4—C2	105.64 (12)	F1—C7—C6	117.94 (16)
C8—C9—C4	119.04 (14)	F1—C7—C8	117.45 (15)
C8—C9—H9	120.5	C6—C7—C8	124.61 (15)
C4—C9—H9	120.5		

### Hydrogen-bond geometry (Å, º)

**Table:**

*D*—H···*A*	*D*—H	H···*A*	*D*···*A*	*D*—H···*A*
N1—H1···O1^i^	0.86 (2)	2.159 (19)	2.8925 (17)	142.8 (17)
O2—H2···O1^ii^	0.82	1.78	2.5954 (17)	170

Symmetry codes: (i) *x*, −*y*+1/2, *z*+1/2; (ii) −*x*+1, −*y*, −*z*.
